# Molecular Dynamics Study of Magnesium and Sodium Ion Transport in a Sulfonated Cation Exchange Membrane

**DOI:** 10.1021/acsomega.6c02058

**Published:** 2026-07-17

**Authors:** María Pérez-Grisales, Iván Moncayo-Riascos, Sergio Castañeda, Carlos Sánchez-Sáenz

**Affiliations:** Departamento de Procesos y Energía, Facultad de Minas, 124148 Universidad Nacional de Colombia, Medellín, Antioquia 050034, Colombia

## Abstract

This study focused on understanding the diffusion of sodium and magnesium ions in a sulfonated poly­(ether ether ketone) cation exchange membrane as a function of water content and ionic composition. For that aim, molecular dynamics simulations were used to represent a conductive environment involving the polymer’s functional chains, water molecules and ions. Mean squared displacement and self-diffusion coefficients revealed subdiffusive behavior, indicating that ion mobility is limited by the membrane’s structure, leading to slower-than-normal diffusion rates. Magnesium ions exhibited self-diffusion coefficients 2 orders of magnitude lower than sodium ions and established stronger interactions with membrane functional groups than monovalent ions, affecting properties like porosity and tortuosity. The water content and polymer structure were critical in conforming hydrophilic networks that support ion mobility and diffusion. This research gives valuable insights into the transport of divalent ions through cation exchange membranes, which is relevant to applications such as electrodialysis and reverse electrodialysis.

## Graphical abstract

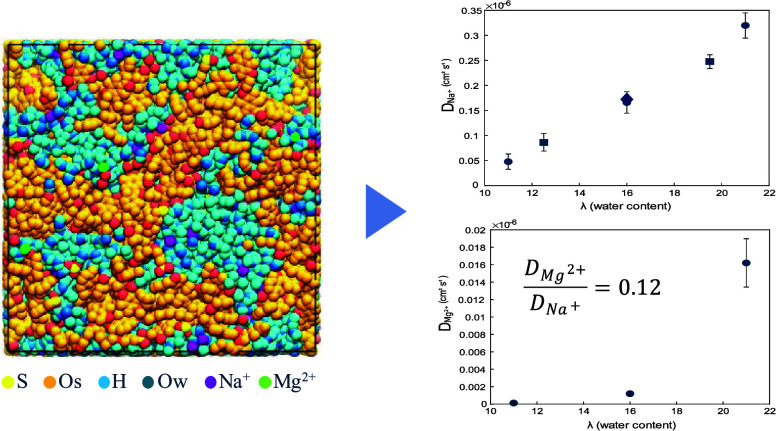

## 1 Introduction

Most of the efforts for the fabrication of new membranes for reverse electrodialysis (RED) applications focus on the exploration of new materials with high conductivity and chemical stability, the development of monoselective membranes, and the innovation of new and better fabrication methods.^1,2^ However, challenges remain in understanding ion transport mechanisms, especially in the presence of multivalent ions.^3,4^ For instance, the Donnan effect, which normally facilitates the exclusion of co-ions to enhance membrane selectivity, becomes less effective. In consequence, the performance of membranes and energy production in a RED device is reduced.^4,5^

Typically, the performance of RED devices has been estimated based on the concentration difference of solutions exclusively containing NaCl, since they dissociate to the ions found in highest proportion in natural waters (e.g., seawater, river water, or brines); however, they also contain multivalent ions that decrease appreciable the amount of energy that can be extracted through RED.^4,6^ In fact, divalent ions are involved in a reduction in the open-circuit potential, the countercurrent transport of counterions, and diffusion coefficients lower in magnitude than monovalent ions. Those effects lead to a decrease in salt gradient energy and, thus, a lower net power density produced using a RED device.

In general, the study of the effects of multivalent ions in the transport through ion exchange membranes has focused on Mg^2+^. On the one hand, they have a strong effect on transport, as mentioned in the preceding paragraph, and are in appreciable quantities compared to other ions.^7^ On the other hand, divalent ions affect cation exchange membranes more severely than anion exchange membranes.^4^ Consequently, a theoretical approach is required to elucidate how multivalent ions affect membrane structure and ion–polymer interactions, and to interpret the resulting interplays between transport-related properties normally difficult to measure and analyze experimentally.^4,8,9^

Classical molecular dynamics (MD) simulations are useful to deepen the understanding of both structural and transport properties of ions in polymeric membranes.^12–16^ They provide detailed insights into ion transport in heterogeneous systems, including polymeric systems, zeolites, swelling clays, and hydrogels, and enable the description of transport phenomena at the molecular scale.^15–19^ Furthermore, these simulations offer detailed information on membrane structure, charge distribution, and ion dynamics, which allows optimizing membrane design and improving performance in practical applications.^16^

Previous MD studies revealed that ion transport in conducting polymers depends on water channels within the membrane.^19–21^ Volkov et al.^21^ found that ionic conductivity is limited by ion transport through narrow channels. The characteristics and pore size of these channels depend on the polymer matrix and the hydration level.^20,21^ As a matter of fact, ionic pairing is more likely at low hydration levels, while at high hydration levels, ion transport is more like to Brownian motion in solution.^20^ Some authors have proposed alternative diffusion mechanisms to study deviations from normal diffusive behavior of ions and water molecules in polymeric matrices. These are based on experimental spectroscopy techniques and computational methods such as MD and ab initio simulations.^10,11,22,23^

Badessa and Shaposhnik^24^ used ab initio simulations to study the selective transport of multicharge cations through ion-exchange membranes in the electrodialysis process. Based on their findings, ionic interactions of multivalent ions play a determining role in the transport of these cations, whereas for monovalent ions hydrogen bonds have a greater influence.^24^ Additionally, Soldatov et al.^25^ remark on the impact of hydrogen bonds and specific interactions between ions and membrane functional groups as key factors in selective ion transport.

Zhang et al.^22^ used molecular dynamics to study the diffusion of Na^+^ and Cl^–^ ions in poly­(styrenesulfonate) and poly­(diallyl dimethylammonium chloride). Their results showed that the ions follow specific trajectories, which move through “water pockets” within the polymeric channels, that lead to an increase in electrostatic trapping. Berrod et al.^23^ combined advanced spectroscopy techniques with molecular dynamics simulations to achieve a better understanding of water dynamics and ion transport through a perfluorosulfonic acid membrane used in fuel cells. The authors showed anomalous diffusive behavior due to confinement at the nanoscale and interactions with fixed-charged groups at the interphase. Rezayani et al.^11^ used molecular dynamics techniques to calculate the anomalous diffusion coefficients of water in sulfonated poly­(ether sulfone) membranes, which showed good agreement with experimental results. This research demonstrates that the morphology of the membrane and its porosity are decisive in ion mobility. Finally, a similar study was conducted for an anion exchange membrane of poly­(phenylene oxide) and quaternary ammonium by Luque Di Salvo et al.^10^ The results showed anomalous diffusion for ions and water molecules, indicating that diffusion significantly depends on the morphology of the hydrophilic channel, particularly the polymer chain morphology and hydration level.^10^ Despite the above, few theoretical investigations have allowed an improvement in the understanding of the transport of divalent ions through ion-exchange membranes.^8–11^

For a better understanding of transport of monovalent and divalent ions across the membrane, different molecular dynamics simulations of a cation-exchange membrane in the presence of Na^+^ and Mg^2+^ ions and different water content were performed, considering that ionic transport and membrane properties are closely related to the polymer structure and morphology when hydrated.^26,27^ Existing studies of ion behavior in polymer ion-exchange membranes have primarily focused on monovalent ions, single-ion systems, and ion-pairing phenomena using theoretical or state-based models rather than atomistic simulations.^28–30^ Although the hydration structure and slow water-exchange kinetics of Mg^2+^ are well documented,^31,32^ to the best of our knowledge, no prior work has directly examined Na^+^/Mg^2+^ transport throughout a polymeric ion-exchange membrane and connected the strong hydration constraints of Mg^2+^ to its mobility and interactions inside these materials. In this work, we address this gap by providing a molecular-level description of Na^+^/Mg^2+^ behavior inside a polymer taking into account solvation structures, local interactions and transport.

The sulfonated poly­(ether ether ketone) (SPEEK) was selected for simulations due to its favorable properties, including ionic conductivity, chemical stability, and mechanical strength, making it a feasible option for ion-exchange applications such as electrodialysis and reverse electrodialysis (RED).^33,34^ This polymer has been extensively studied in both theoretical and experimental research for various applications, including desalination and energy storage systems.^23–25^ In this work, ab initio techniques were employed to optimize the SPEEK monomer structure, which was subsequently used to construct hydrated membrane models for molecular dynamics simulations.^35^ The transport behavior of Na^+^ and Mg^2+^ ions was investigated under different hydration levels and ionic compositions through the analysis of local ion distribution, solvation effects, interaction energies, pore structure, and diffusion behavior. The results establish molecular-level relationships between hydration, membrane microstructure, and ion transport, providing insight into the factors governing divalent-ion limitations in hydrated ion-exchange membranes.

## 2 Methods

### 2.1 Membrane Model

The SPEEK monomer was obtained via ab initio techniques, starting from three constitutive structures (see Figure 1). The resulting optimized monomer was subsequently used to construct the initial polymer segment for the MD simulations. The hydrated membrane model for the MD simulations was generated by randomly inserting 216 polymer segments (two SPEEK subunits and one PEEK unit), ions, and water molecules using the PACKMOL software. This composition corresponds to a degree of sulfonation of ∼66.7%, which lies within the range of experimentally reported DS values for SPEEK membranes with acceptable stability and is close to values reported as optimal in a representative RED study (∼65%), where the highest power density was obtained.^33,36–38^ Next, an equilibration step was performed, moving all atoms in the system. Following equilibration, the short SPEEK segments were interconnected to form a continuous macromolecular network using a distance-based automated bonding procedure implemented in VMD.^39^ A production run was then conducted, treating the polymer as a solid through which ions and water molecules can move. The resulting computational membrane model was considered suitable for analyzing the differences in ion transport between divalent and monovalent ions and their relation to the polymer structure for different hydration levels and ionic compositions.

**Figure 1:**
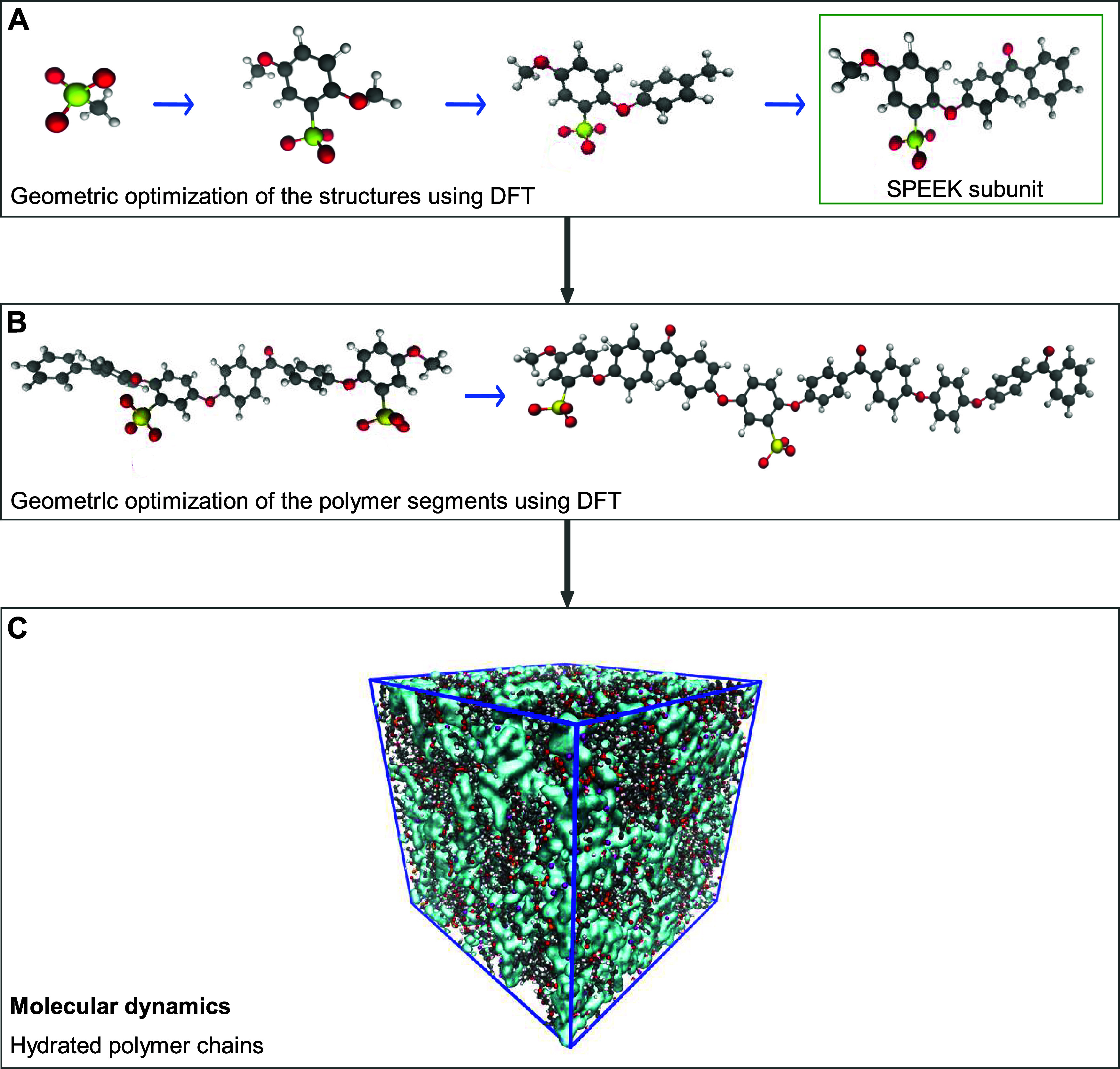
Graphical representation of the simulation box: (A) Constitutive structures of the SPEEK monomer. (B) Structures of the SPEEK two-monomer and three-monomer polymer segments. (C) Simulation box equilibrated by molecular dynamics. The carbon atoms are represented by gray color, hydrogen white, oxygen red and sulfur yellow. Water is represented cyan.

The structure obtained from the previous procedure was further equilibrated during molecular dynamics simulation to obtain a more realistic representation (see Section 2.3). Finally, the number of water molecules in each case and the composition of Mg^2+^ and Na^+^ ions are presented in Table 1. To represent the ionic composition in a simplified way, the graphs include the Mg^2+^/Na^+^ concentration ratio for each system.

**Table 1:** Compositions of Membrane Models Used in Molecular Dynamics Simulations

system	box size (nm)	hydration level (λ)	water uptake (%)	Na^+^ (mol/L)	Mg^2+^ (mol/L)
S_1_	8.045	16	53	1.38	0
S_2_	7.799	12.5	41	1.17	0.10
S_3_	8.266	19.5	65	1.17	0.10
S_4_	7.679	11	36	0.69	0.34
S_5_	8.028	16	53	0.69	0.34
S_6_	8.350	21	70	0.69	0.34

The hydration levels (λ = 11–21) correspond to water uptake values that fall within the range typically reported for SPEEK membranes in experimental studies. For instance, water uptake values up to ∼ 80% have been reported, depending on the degree of sulfonation (DS).^33,40–42^ Specifically, SPEEK membranes with DS% between 59.15–71 exhibit water uptake values ranging from 19.44% to 86.79%.^40^ Furthermore, another study reported water uptake values between 25% and 81% for DS% from 57 to 87.^42^

In the context of reverse electrodialysis applications, membranes exposed to saline solutions such as NaCl swell until reaching an equilibrium state. The degree of swelling of a SPEEK membrane in direct contact with water depends on various factors, including the membrane fabrication protocol, the degree of functionalization, ionic concentration, and the valence number of the ions.^42–45^ Increased water content enhances ion mobility and conductivity, but excessive swelling may compromise structural integrity and selectivity in RED applications. Therefore, the selected value falls within the range experimentally reported in the literature, corresponding to moderate to high water uptake values.^42^

The polyelectrolyte model was represented using the OPLS-AA force field, which has demonstrated good accuracy in modeling polymers and ion-exchange materials,^22,46,47^ and has been successfully applied to predict the physical properties of polyimides,^48^ poly­(ethylene oxide),^46^ and polyelectrolyte assemblies such as poly­(sodium-4-styrenesulfonate) and poly­(diallyldimethylammonium chloride).^22^ The explicit SPC/E model was used to represent water molecules as rigid bodies with three interaction sites. These sites correspond to hydrogen atoms with positive partial charges and oxygen atoms with negative partial charges.^49,50^ The Lennard-Jones potential (LJ) was used to model van der Waals interactions between ion–ion, ion–molecule, and molecule–molecule pairs as shown in eq ([Disp-formula eq1])

1 $$E_{\mathrm{LJ}}=4\varepsilon_{\mathrm{ij}}^{*}\left[{\left(\frac{\sigma_{\mathrm{ij}}^{*}}{r_{\mathrm{ij}}}\right)}^{12}-{\left(\frac{\sigma_{\mathrm{ij}}^{*}}{r_{\mathrm{ij}}}\right)}^{6}\right]$$

where ε* is the depth of the potential well and σ* is the intermolecular separation when the potential energy is zero. The parameters σ*_ij_* and ε*_ij_*, were estimated from the geometric mixing rule, as shown in eqs ([Disp-formula eq2]) and ([Disp-formula eq3])­

2 $$\sigma_{\mathrm{ij}}=\sqrt{\sigma_{i}\sigma_{j}}$$

3 $$\varepsilon_{\mathrm{ij}}=\sqrt{\varepsilon_{i}\varepsilon_{j}}$$

The simulation of the ions was carried out under the approximation of point charges, so the Coulombic interactions were calculated by eq ([Disp-formula eq4])

4 $$E_{\mathrm{coul}}=\frac{q_{i}q_{j}}{4\pi \epsilon r^{2}}$$

The parameters for the SPC/E model and the Lennard-Jones potential are displayed in Tables S1 and S2, respectively, of the Supporting Information.

It is worth noting that the results reported in this study depend on the system size and the molecular distribution characteristics. Therefore, density and potential energy were used as criteria to verify an appropriate equilibration of the systems and model consistency. The calculated density was compared with the density of the membrane/water system using the semiempirical equation implemented by Weber and Newman^51^

5 $$\rho =\frac{\mathrm{EW}+M_{0}\lambda }{{\mathrm{V̅}}_{m}+\lambda {\mathrm{V̅}}_{0}}$$

where EW is the equivalent weight of the membrane, *M*_0_ is the molecular weight, λ is the hydration level of water (number of water molecules per membrane functional group), *V̅*_0_ is the partial molar volume of water, and *V̅*_*m*_ is the partial molar volume of the dry membrane (ρ_dry_), which is calculated as follows

6 $${\mathrm{V̅}}_{m}=\frac{\mathrm{EW}}{\rho_{\mathrm{dry}}}$$

A density of 1.241 g·cm^–3^ was used for the dry membrane, in agreement with the experimental value reported in the literature.^52^

### 2.2 Simulation Details

Molecular dynamics simulations were performed at a temperature and pressure of 298 K and 1 atm, respectively (typical operating conditions at the RED device), using LAMMPS free software for MD simulations. Periodic boundary conditions were applied in all dimensions. An energy minimization was conducted (total energy tolerance of 1.0 × 10^–6^ kJ mol^–1^), followed by an equilibration step of the system during 15 ns, applying both the NPT and NVT ensembles. The NVT ensemble (canonical ensemble) maintains a constant number of particles, fixed volume, and temperature through a thermostat, whereas the NPT ensemble (isothermal–isobaric ensemble) maintains a constant number of particles, pressure, and temperature by using both a thermostat and a barostat.

The NPT ensemble was used for 5 ns, followed by the NVT ensemble for 10 ns, with a time step of 1 fs. Temperature and pressure were kept using the Nosé–Hoover thermostat and barostat, respectively. A damping coefficient of 100 time steps was used for the Nosé–Hoover thermostat, while a damping coefficient of 1000 time steps was used for the barostat. A cutoff radius of 1.6 nm was applied for short- and long-range interactions. The long-range interactions were calculated using the PPPM method with a precision of 1.0 × 10^–4^, and the equations of motion were integrated using Verlet’s algorithm with a time step of 1.0 fs.

The VMD software was used to recalculate the bonds, angles formed by three atoms, and dihedral and improper angles formed between four atoms for the final configuration obtained after the equilibration stage. This configuration resulted in polymer structures exhibiting apparent entanglement, as well as a uniform distribution of water molecules and ions. These structural features arise due to molecular interactions that take place within the system. Also, this final configuration was used to evaluate the diffusion of ions in the membrane. The polymer was immobilized for this evaluation, considering that the membrane dynamics are much slower than that of the ions and water molecules. Finally, a production step was carried out using an NVT ensemble for 10 ns with a time step of 1 fs.

### 2.3 Calculation of Self-Diffusion Coefficients

The self-diffusion coefficients of the ions were calculated from the mean squared displacement (MSD).^53^

7 $$\mathrm{MSD}=\sum_{i=1}^{N}\left\langle r_{i}\left(t\right)-r_{i}\left(0\right)\right\rangle$$

where *r_i_* (*t*) is the position of particle *i* at time *t* and *r_i_* (0) is the initial position of the particle. From eq ([Disp-formula eq5]) and the Stokes–Einstein equation, the self-diffusion coefficient is calculated using the following equation^53^

8 $$D=\frac{1}{6}\lim_{t\to \infty }\frac{d\left(\mathrm{MSD}\right)}{dt}$$

The heuristic commonly used to verify normal diffusion is to check that the slope of the log­(MSD) versus log­(*t*) plot is approximately 1.0.^54^

### 2.4 Radial Distribution Functions and Coordination Number

The radial distribution function (RDF) was used to analyze the structure of the system and the distribution of counterions around the functional groups of the polyelectrolyte. It also helps to identify specific interactions between relevant groups and pairs of atoms in the system. The RDF between atom pairs *A* and *B* is calculated as follows^53^

9 $$g_{\mathrm{AB}}\left(r\right)=\frac{1}{4\pi \rho_{N}N_{B}}\sum_{i\in A}^{N_{A}}\sum_{i\in B}^{N_{B}}\frac{\delta \left(r_{\mathrm{ij}}-r\right)}{r^{2}}$$

where ρ_*N*_ is the average density of atoms, *N* is the total number of atoms, ***r*** *_ij_* is the distance between atoms *A* and *B*, and δ­(***r***_*ij*_ – ***r***) is the Dirac delta function. Finally, the coordination number was calculated as the number of atoms within a spherical volume of diameter *D* around a central reference atom. The cutoff distance *D*/2 used to compute the coordination number was taken as the position of the first minimum of the corresponding RDF after its first peak^53^

10 $$\mathrm{CN}=4\pi \rho \int_{0}^{D/2}r^{2}g\left(r\right)dr$$

## 3 Results and Discussion

### 3.1 Equilibration

The potential energy showed no significant deviations during the last 1500 ps simulation time (see Figure S1 in the Supporting Information). For instance, there was good agreement between the calculated density and the theoretical density estimated from eq 5. The calculated average density was 1.217 g·cm^–3^ for λ = 11, representing an increase of 4.5% compared to the theoretical density, which is 1.165 g/cm^3^. The average density obtained in each system was also consistent with previous studies for the hydrated SPEEK polymer.^55,56^ The consistency between the results shows that the choice of models and methods is appropriate. Nevertheless, it is worth noting that increasing the system size and chain length could enhance the molecular-level details captured by the MD simulations.

### 3.2 Hydrated Membrane Morphology

Figure 2 shows the simulation boxes after the equilibration step. The hydration of the SPEEK membrane leads to the formation of two distinct phases mainly. The hydrophobic phase primarily consists of the polymer structure with sulfonate groups exposed to a hydrophilic phase that contains water molecules and dissolved ions. An increase in water content promotes a more interconnected network of water molecules, as seen in the systems with higher hydration levels (S3 and S6). In contrast, at lower hydration levels (systems S2 and S4), water clusters keep more segregated throughout the membrane.

**Figure 2:**
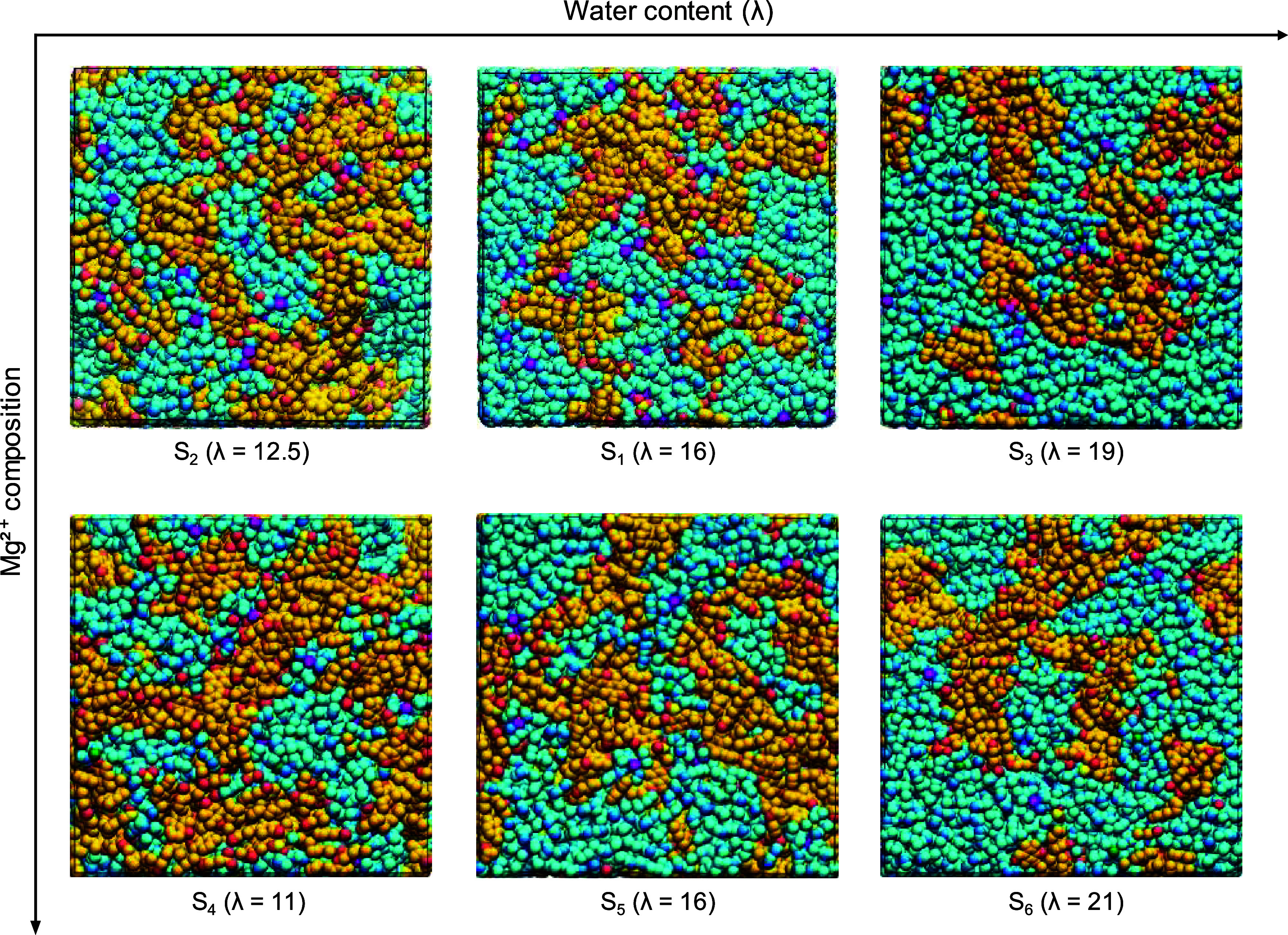
Segregation of the nanophases by varying the water content λ and ion concentration. The ochre color represents the hydrophobic phase of the polymer. The sulfur atoms are represented in yellow, and the oxygen atoms of the sulfonate group are in orange. The hydrogen and oxygen atoms of the water dark blue, respectively. Sodium ions are shown in purple, and magnesium in green.

### 3.3 Distribution of the Membrane Functional Groups

The *g*(*r*)_S–S_ corresponding to the RDF of the sulfur atoms in the sulfonate groups (see Figure 3) provides information about the spatial distribution of the membrane functional groups as the degree of hydration increases and the cation composition (Na^+^ and Mg^2+^) changes. In Figure 3, the position of the highest peak indicates the most probable distance between the sulfur atoms of the functional groups (S–S), which changes with respect to the hydration level and the composition of ions in the system. For most graphs, the S–S distance was between 0.675 and 0.685 nm. By contrast, at highest hydration levels (λ = 19.5 and 21), the first peak is less pronounced and the maximum values of the RDF curve range between 0.845 to 0.865 nm. This change reflects an increased water content on the spatial distribution of the interacting atoms. The observed distances correspond to those reported in previous experiments for SPEEK based polymers, which values oscillate between approximately 0.7 to 1.2 nm.^57,58^

**Figure 3:**
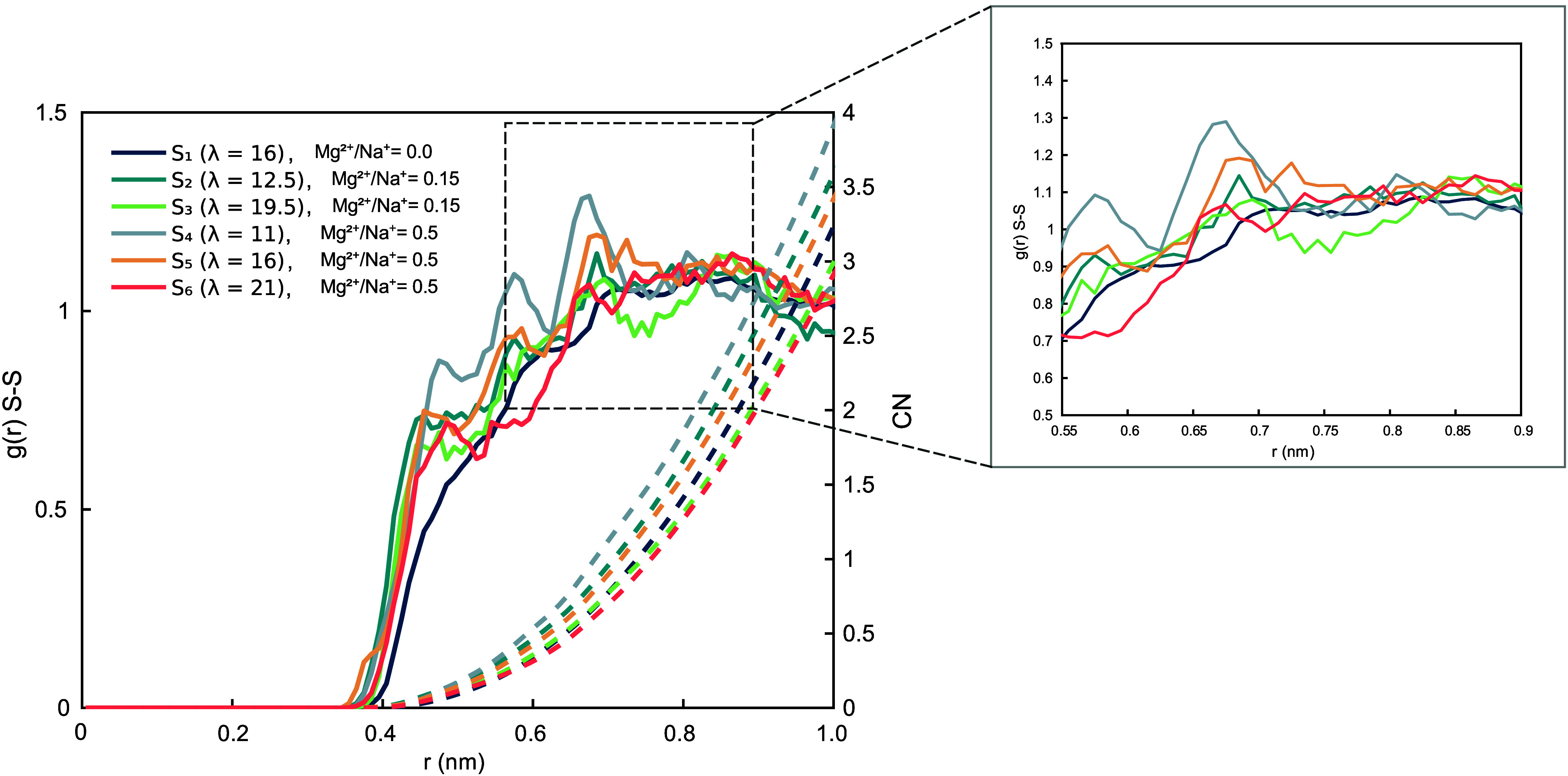
RDFs for sulfur–sulfur atoms of the sulfonic acid group at different hydration levels. The inset on the upper right highlights the predominant peak for better visualization of the information.

The hydrophilic channels increase in solvation and swelling as water content goes up, resulting in a higher separation of the functional groups than system with low water content. At the highest hydration level (λ = 21), a sharp decrease in the height of the predominant and neighboring peaks takes place. A reduction of the interactions between neighboring functional groups due to increased water content. These observations correspond with results reported by authors that studied SPEEK or similar systems.^56,59,60^ As the water content in the membrane increases, functional groups become more separated, leading to a decrease in coordination between the representative atoms of these groups.^45^ Additionally, the high water content in the membrane implies drastic changes in the conformation of the SPEEK polymeric chains.^61^

In the absence of Mg^2+^ (see system S_1_), there is no predominant peak suggesting that electrostatic interactions involving divalent ions and sulfonate groups could be sufficiently strong to produce a compaction of local chains. This latter is related to the higher ability of magnesium ions than sodium ions to coordinate with multiple functional groups as found in.^62^ In contrast, sodium ion establishes weaker interactions than magnesium with the functional groups due to its low effective charge, which results in a more homogeneous charge distribution in the membrane and a lower tendency to conform ordered structures than divalent ions, as described in.^24,63^

Coordination numbers (CN) between sulfur atoms (S–S) reflect the distribution of sulfonate groups with respect both water content and ionic composition. As hydration increases, the coordination number decreases, indicating a more dispersed arrangement of the sulfonate groups. This dispersion occurs because polymer matrix swelling goes up as new water molecules are added to the system, increasing the distance between neighboring sulfonate groups.

The solvation of the membrane functional groups was analyzed from the distribution of water molecules around the sulfur atom (*g*(*r*)_S–OW_) and the oxygen atom with the negative partial charge of the sulfonate group (*g*(*r*)_OS–OW_). This is shown in Figure 4 for different hydration levels. Both RDFs exhibit two peaks: the first (with higher intensity than the second) corresponds to the first solvation shell, while the second corresponds to the second solvation shell. In particular, Figure 4-A, which corresponds to *g*(*r*)_S–OW_, displays a first peak at an approximate distance of 0.385 nm, whereas for *g*(*r*)_OS–OW_ in Figure 4-B, the peak of highest intensity is located at 0.265 nm. In both cases, increasing the water content in the membrane increases solvation. This reduces the direct interactions between functional groups and other water molecules. These observations are consistent with results reported by other authors for similar membrane systems.^11,13,64,65^ Furthermore, as water content increases, the coordination number of the oxygen atom of the sulfonate group shows a slight increase. This suggests that the coordinated structure of the sulfonate group remains relatively stable regardless of hydration levels and ionic compositions.

**Figure 4:**
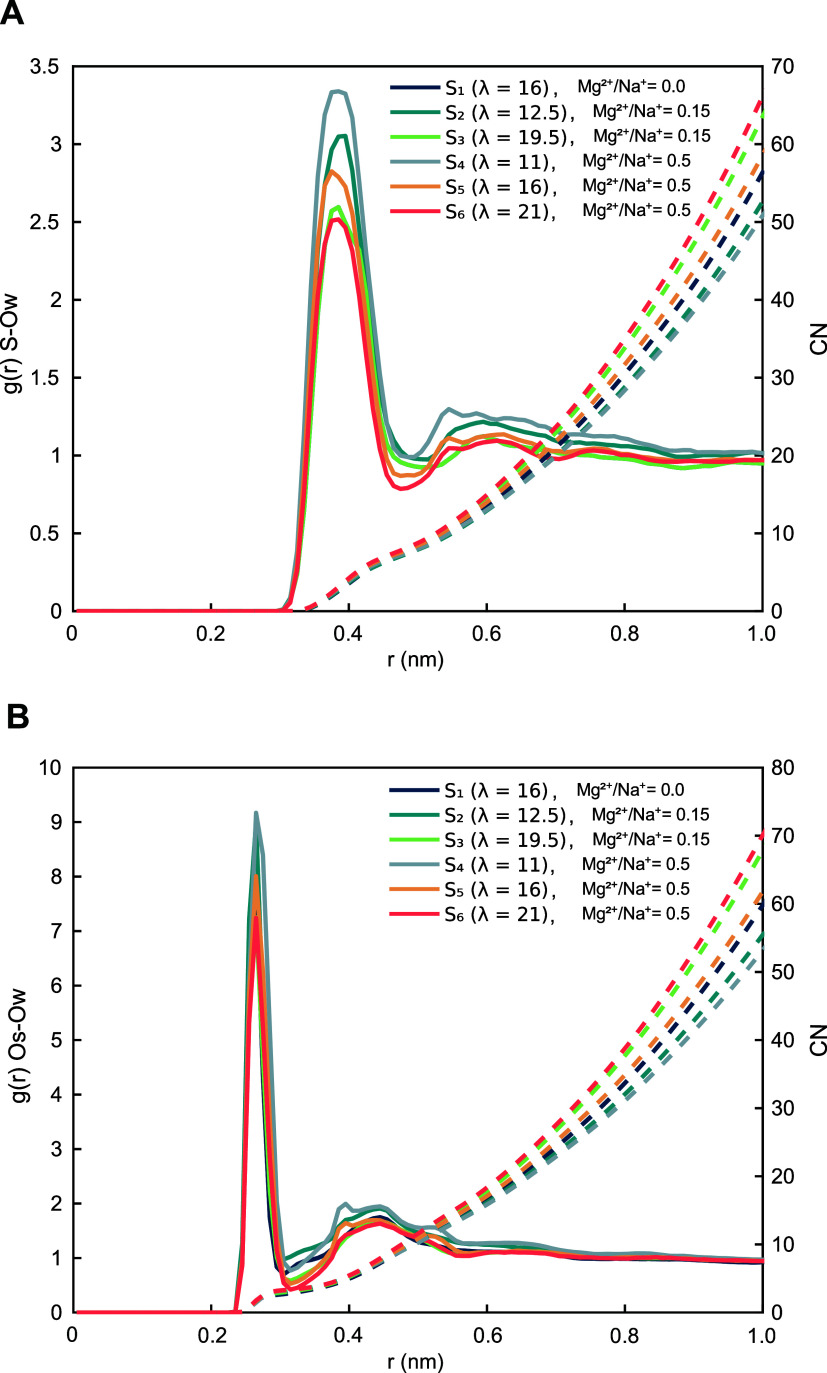
RDF for different hydration levels and compositions. (A) Between the sulfur atom of the functional group and the oxygen atom of the water molecule. (B) Between the oxygen atom with the negative partial charge of the functional group and water.

The distribution and relative positions of the functional groups in the membrane influence ion transport and the selectivity between divalent and monovalent ions. In particular, when the distance between functional groups is relatively high, the selectivity for monovalent ions increases, i.e., these ions are more likely to condense onto the membrane. Conversely, when anionic groups are closer together, a higher affinity for divalent ions is observed.^66,67^

### 3.4 Effects of Ion Solvation

The radial distribution functions *g*(*r*)_Na^+^−OW_ and *g*(*r*)_Mg^2+^−OW_ provide insights into the spatial distribution of water molecules around cations. Figure 5 shows *g*(*r*)_Na^+^−OW_, corresponding to sodium and the oxygen of water molecules. The first peak appears at 0.235 nm, with its height ranging from 18.58 at λ = 11 to 15.04 at λ = 21, indicating a slight decrease in ion–water structure as hydration increases. In contrast, *g*(*r*)_Mg^2+^−OW_ exhibits a much higher first peak than *g*(*r*)_Na^+^−OW_, with values between 31.23 (λ = 12.5, S_2_) and 38.63 (λ = 19.5, S_3_). This peak shifts from 0.195 nm at the lowest hydration levels (λ = 11 and 12.5) to 0.205 nm at the highest hydration levels (λ = 16–21). These results suggest that magnesium ions form stronger and more structured interactions with surrounding water molecules compared to sodium ions. The higher intensity of the first peak in *g*(*r*)_Mg^2+^−OW_ confirms the higher relative probability of finding water molecules in the immediate hydration shell of Mg^2+^.

**Figure 5:**
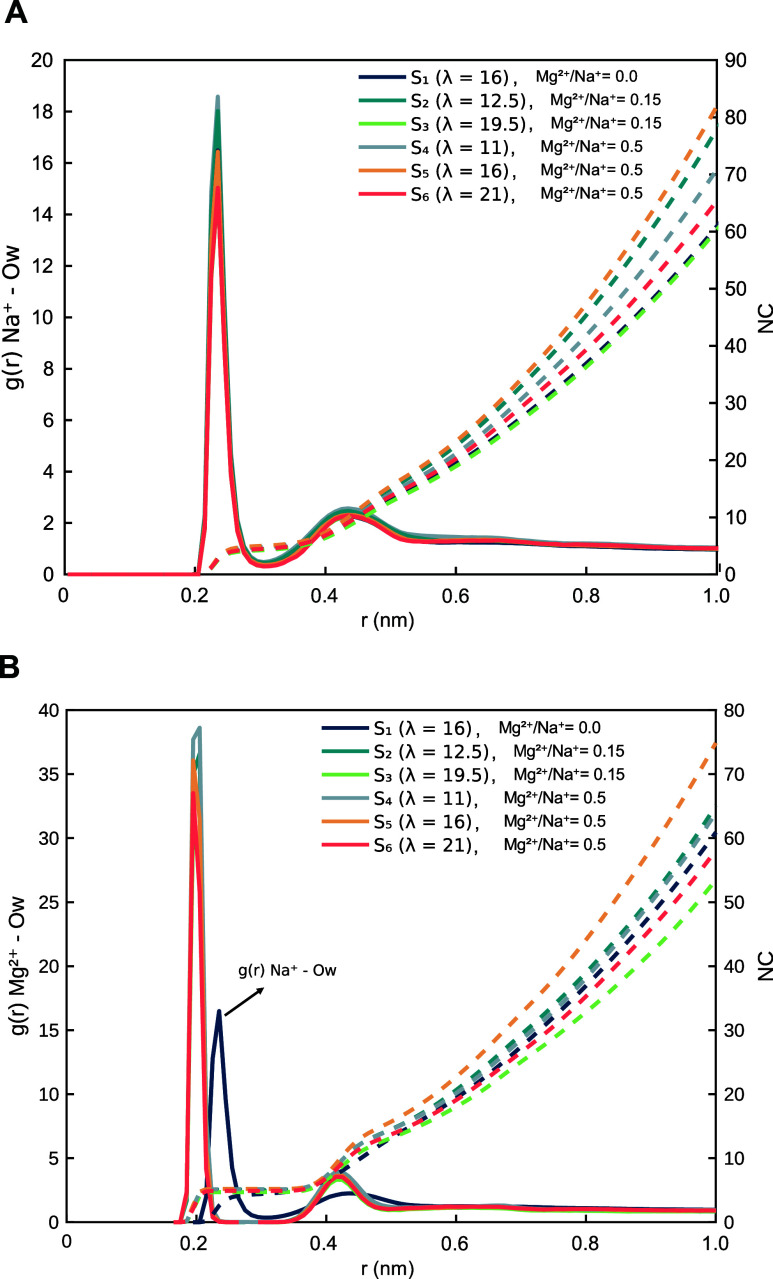
RDF for different hydration levels and compositions. (A) Between the sodium ion and the oxygen atom of the water molecule. (B) Between magnesium ion and water.

Additionally, the analysis of the radial distribution function *g*(*r*)_Mg^2+^−OW_ shows that, after the first peak, the probability of finding a water molecule near the magnesium ion decays to zero. This behavior suggests the formation of well-defined coordination complexes between Mg^2+^ and its surrounding water molecules. It is important to indicate that other significant effects, such as polarization, are neglected when ions are treated as point charges. These effects can strongly influence charge distribution in solution, particularly in the presence of divalent ions. Therefore, further accuracy of the obtained result could be achieved by considering more sophisticated models capable of capturing solvent polarization, as well as ion–solvent and ion–polyelectrolyte interactions.

These solvation effects also influence the magnitude of the interaction between the functional groups and the cations, as shown in the RDFs between the ions and the oxygen atom of the sulfonate group (Figure 6). For both *g*(*r*)_Na^+^−OS_ and *g*(*r*)_Mg^2+^−OS_, the intensity of the first peak decreases as the hydration level increases. At the lowest hydration level (λ = 11), the sulfonate group interacts stronger with cations than at higher hydration levels. Also, the cations are located closer to the membrane functional groups than at the highest hydration level. Even more, as the water content increases, solvation of the ions reduces the attractive electrostatic forces of the sulfonate groups on the ions. In particular, the first peak in the *g*(*r*)_Na^+^−OS_ function is located at 0.225 nm, with a height varying from 27.77 for λ = 21 to 52.85 for λ = 12.5. For the *g*(*r*)_Mg^2+^−OS_ function, the first peak shifts to a shorter distance of 0.185 nm compared to *g*(*r*)_Na^+^−OS_, and has a larger height than for sodium (approximately four times), ranging from 142.81 to 213.77.

**Figure 6:**
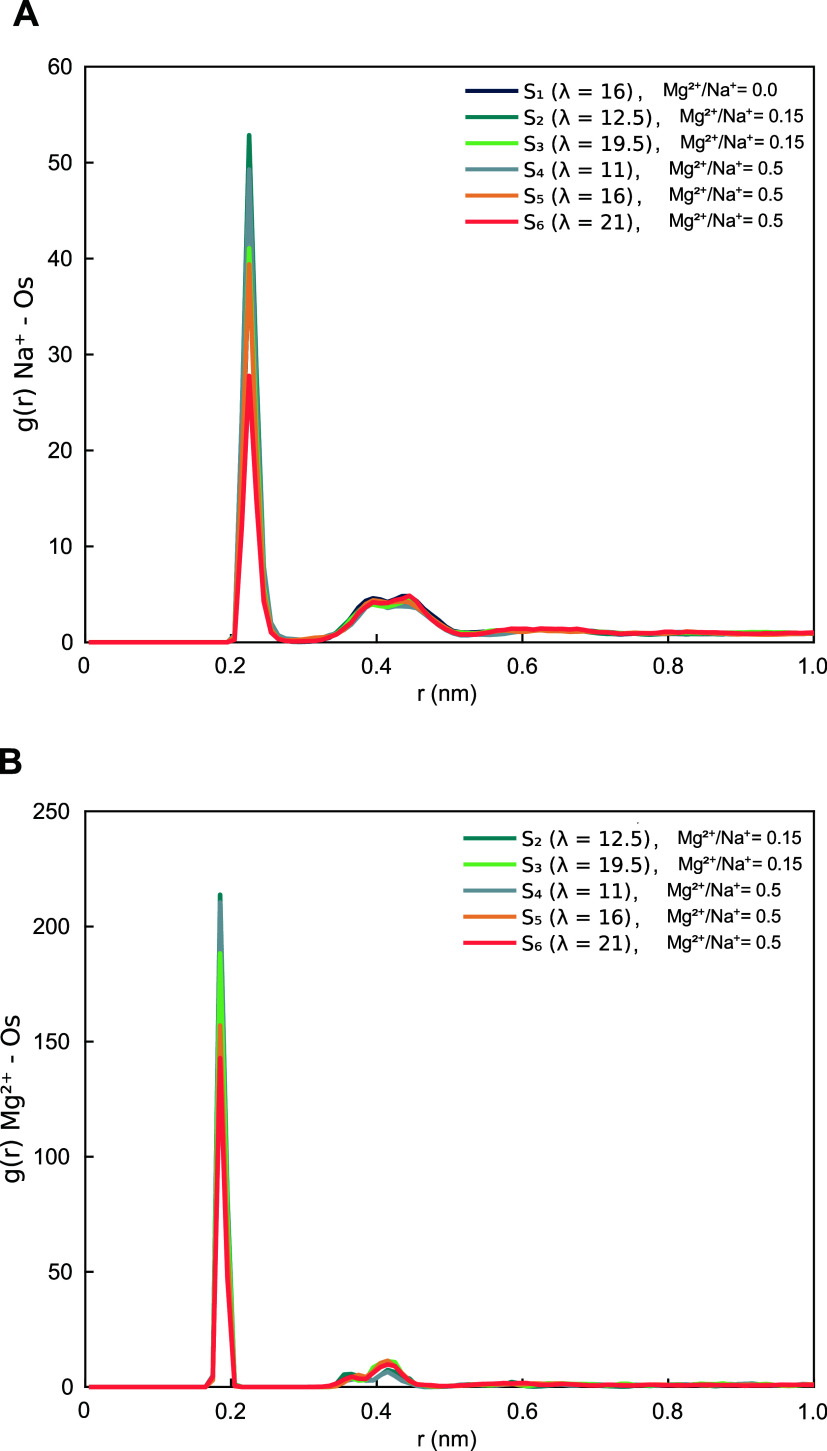
RDF for different hydration levels and compositions. (A) Between the sodium ion and the oxygen atom of the sulfonate group. (B) Between the magnesium ion and the oxygen atom of the sulfonate group.

Moreover, for both ions, a region where the function becomes zero is observed after the first peak, suggesting the formation of strong interactions that could lead to the formation of ionic pairs. In the specific case of magnesium, the region where the probability is zero is more extensive than for sodium, indicating a higher propensity of magnesium to interact with the functional groups and form ionic pairs. The strong association between divalent ions and membrane functional groups directly influences ionic conductivity and permselectivity by neutralizing part of the membrane charge.^4^

An increase in the coordination number is observed due to enhanced ion solvation, which aligns with the reduction in RDF peak intensity between cations and water molecules (see Figure 6). This reduction indicates that additional water molecules in the hydration shell establish less structured bonds with the cations. For Na^+^, the coordination number increases from 4.3 at λ = 11 to 5 at λ = 21, calculated within a radius of 0.305 nm corresponding to the first hydration layer. In contrast, Mg^2+^ shows more significant variations, with its coordination number increasing from 4.7 to 5.2 as water content rises (λ = 19.5 and 21), calculated within 0.275 nm. The stronger electrostatic interactions of the divalent magnesium ion with water molecules result in a more compact hydration shell. This effect is well documented in the literature, in which smaller cations with higher charge densities, such as Mg^2+^, exhibit stronger Coulombic attraction to surrounding water molecules, leading to higher coordination numbers and a more structured hydration shell compared to larger, monovalent cations like Na^+^.^68,69^

A higher coordination number for magnesium than sodium implies that magnesium is able to establish more interactions. In consequence, the membrane requires a higher energy to break the interactions between magnesium and binding sites and to form new interactions as the ions move throughout the membrane. Additionally, Mg^2+^ has a much lower hydration free energy (−1830 kJ·mol^–1^) compared to Na^+^ (−365 kJ·mol^–1^), indicating that it is more difficult for the magnesium ion to separate detach from the water molecules in its hydration layers and bind to the charged sites on the membrane.^67^

These results are supported by the interaction energy between the ions and the membrane functional groups, as shown in Figure 7. This energy was determined by adding the nonbonded van der Waals and Coulombic interactions between the cations and the functional groups of the polymer. To ensure consistency in the analysis, the total interaction energy was normalized by dividing it by the number of functional groups in the polymer. In this context, negative energy indicates an attractive interaction between the cation and the anionic group. Systems from S_2_ to S_6_ include both counterions, which allows a direct comparison of their interactions with the functional groups. The larger interaction energy magnitude for Mg^2+^ relative to Na^+^ confirms that Mg^2+^ exhibits a strong interaction with the SO_3_^–^ group. Furthermore, increasing water content in the membrane decreases the interaction energy between cations and anions, consistent with previous observations in Figures 5 and 6. This decrease in interaction energy impacts ion transport across the membrane, promoting lower activation energy for diffusion and increasing transport numbers. It also affects the partitioning of ions in the membrane. During partitioning, the divalent ion has a greater tendency to distribute within the membrane matrix due to its charge and strong interactions with the anionic functional groups. This difference influences the selectivity of the ion-exchange membrane for these two ions and its ability to selectivity transport magnesium or sodium across the membrane.

**Figure 7:**
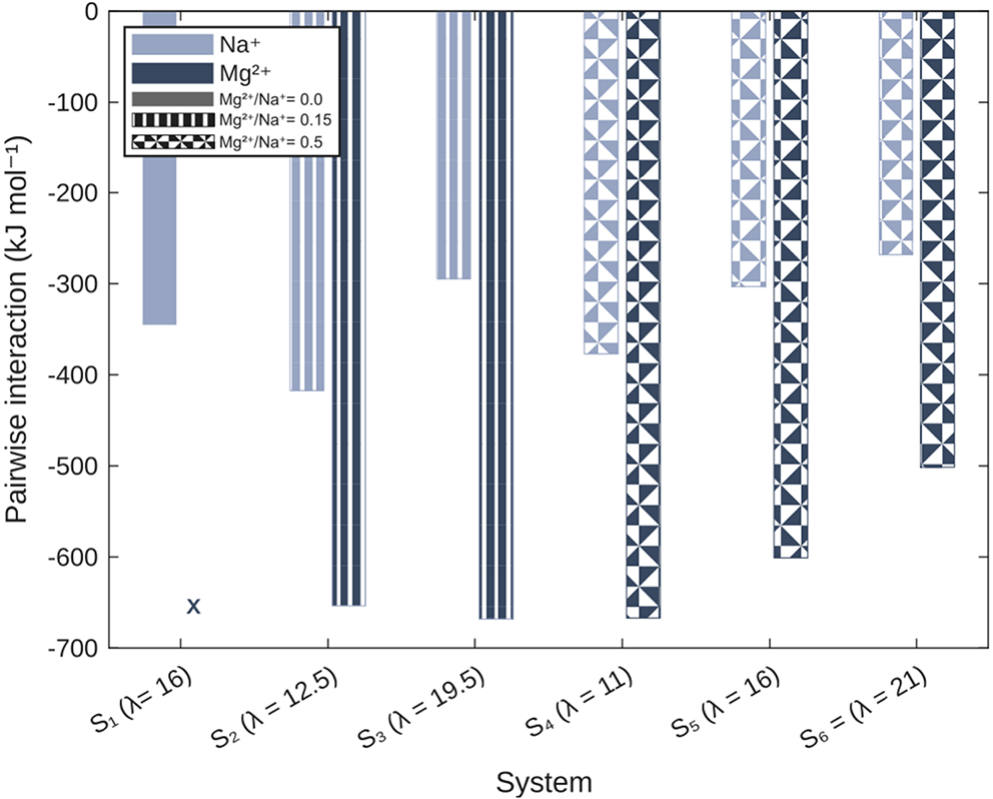
Pairwise energy for each system between the cations and sulfonate groups: Na^+^–SO_3_^–^ and Mg^2+^–SO_3_^–^.

### 3.5 Counterions Diffusion

The MSD analysis reveals significant differences in ion mobility (see Figures S3 and S4 in the Supporting Information). Na^+^ exhibits up to 2 orders of magnitude higher MSD values compared to Mg^2+^. As the water content increases, the system properties tend to resemble those observed in solution. The MSD curves for magnesium ions at the lowest concentrations were excluded from the analysis, since the limited number of atoms can lead to large fluctuations in individual positions and thus increase the uncertainty of MSD determination. In addition, the condition for normal diffusion was verified by checking the linearity of the log–log plot of MSD­(t), where *n* ≈ 1 corresponds to Brownian diffusion. Deviations from linearity, indicative of subdiffusive behavior (*n* < 1), were observed, suggesting that MSD ∼ *Dt^n^*.^56,57^ Although MSD curves do not display strict linearity across all time scales, diffusion coefficients were extracted from the long-time regime, where a stable linear trend emerges.

Sodium ions exhibited mild subdiffusive behavior (0.7 < *n* < 1), approaching normal diffusion (*n* ∼ 1) at higher hydration levels (λ = 16–21). In contrast, magnesium ions displayed pronounced subdiffusive behavior (*n* < 0.7), except at the highest hydration level (λ = 21). In both cases, a transition toward more anomalous diffusion was observed as the water content decreased. The MSD curves did not show uniform behavior across all time scales. At short times, ion motion was restricted by interactions with the polymer and the membrane structure. These restrictions were stronger at low hydration levels, where ions experienced greater difficulty moving freely. Over longer times, ions gradually overcame these restrictions, and their motion became more consistent with normal diffusion. Such behavior is common in polymeric membranes, where the heterogeneous structure hinders ion and molecule transport.^10,11,22,23^ Furthermore, the strong interactions of divalent ions with both water and the polymer explain the reduced mobility and the larger deviations from normal diffusion.

The anomalous diffusion exponents are shown in Figure S5 of the Supporting Information. The observed subdiffusion (*n* < 1) indicates that the motion of ions and water molecules across the membrane does not follows a linear progression with time. Instead, their movement is characterized by constraints and intermittent jumps, arising from the polymer structure and its interactions with water and ions. This behavior is consistent with findings from other studies on highly heterogeneous media such as polymers, gels, and biological tissues.^10,11,22^ The limitations to diffusion in these systems are not solely due to physical barriers; strong adhesion regions or specific binding sites can also immobilize particles, increasing the energy required for ions to move. This effect is particularly pronounced for divalent ions, which exhibit stronger interactions with the polyelectrolyte compared to monovalent ions, as indicated by RDF results in the previous section, thereby raising the energy barrier and reducing diffusion rates. Regarding anomalous ion diffusion, Zhang et al.^22^ suggested that the motion of Na^+^ ions is governed by the polymer microenvironment. Its transport combines normal diffusion with a “jumping” mechanism between discrete sites in the polymeric matrix, driven by electrostatic interactions.

In general, for high hydration levels, the slope of the log–log MSD curve approaches unity. Recently, some authors have considered that results between 0.8 and 1 for *n* are satisfactory in simulations of hydrated membranes, and therefore, one can directly apply Einstein’s relation.^10,13^ However, considering that in some cases *n* is far from unity for the Mg^2+^ ion and to compare the diffusive processes between ions, the effective diffusion coefficient was calculated according to the following generalized Einstein equation

11 $$\frac{n}{t}\mathrm{MSD}=6D_{\mathrm{eff}}$$

where *D*_eff_ is the effective diffusion coefficient of the ions, as shown in Figure 8. The effective diffusion coefficient increases as the membrane’s water content rises. This tendency reflects the progressive solvation of the sulfonate groups and the enhanced dissociation of ions into the aqueous phase. As shown in Figure 8A, Na^+^ exhibits a gradual increase in diffusion with hydration, whereas in Figure 8B, Mg^2+^ mobility is strongly hindered at low hydration levels (λ = 11–16). *D*_Mg^2+^_ is approximately between one and 2 orders of magnitude lower than *D*_Na^+^_ depending on the water content. This behavior can be attributed to the stronger electrostatic interactions between Mg^2+^ and the sulfonate groups, compared to those of Na^+^. In this regard, a previous study reported that Mg^2+^ mobility is approximately 1 order of magnitude lower than that of Na^+^ in cation-exchange membranes with low to moderate water content (volume fractions below 0.6).^70^ They also found that Mg^2+^ mobility is significantly more sensitive to membrane hydration, reflecting its higher requirement for maintaining a stable hydration shell. This observation is consistent with the trend identified in this work, where Mg^2+^ diffusion exhibits a stronger dependence on water content, with a more pronounced reduction at low hydration levels compared to Na^+^.

**Figure 8:**
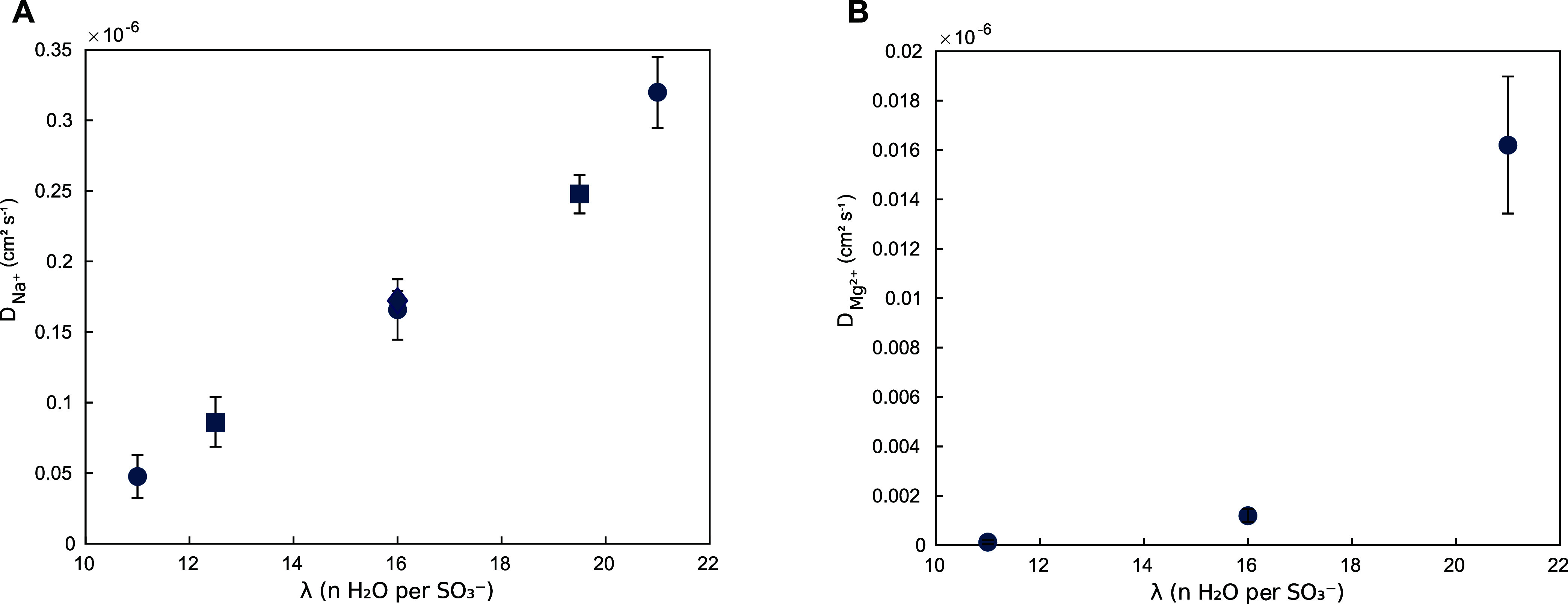
Self-diffusion coefficient of the counterions: (A) Na^+^ and (B) Mg^2+^. In the graph, the different geometrical figures represent the Mg^2+^/Na^+^ concentration ratio for each system: ⧫ (0.0), ■ (0.15) and ● (0.5).

The magnitude of the Na^+^ effective diffusion coefficient obtained in this work is consistent with previously reported values for cation exchange membranes. In single-ion studies of Nafion 117, reported Na^+^ self-diffusion coefficients range from 0.50 to 1.03 × 10^–6^ cm^2^/s, depending on the measurement technique: 0.50–0.55 × 10^–6^ cm^2^/s from pulsed-field-gradient NMR and spin–lattice relaxation,^71^ and 1.0–1.03 × 10^–6^ cm^2^/s from radiotracer experiments.^72,73^ In addition, higher values, up to 2.1 × 10^–6^ cm^2^/s, have been reported for CR61 membranes based on indirect sorption, permeability, and ionic conductivity measurements.^17^

When considering ion mixtures, the Na^+^ tracer diffusion coefficient tends to be lower, ranging from 0.1 to 1.5 × 10^–6^ cm^2^/s, depending on the surrounding counterion and following the trend Mg^2+^ < Li^+^ < Na^+^ < K^+^.^70^ According to the authors, ions move by exchanging positions; thus, the lower mobility of Mg^2+^ limits Na^+^ diffusion.^70^ Furthermore, experimental characterization of seven commercial membranes in the same study reported Mg^2+^ self-diffusion coefficients in single electrolytes between 0.017 and 0.26 × 10^–6^ cm^2^·s^–1^, depending on the water volume fraction (0.3–0.7).^70^ The Mg^2+^ effective diffusion coefficients reported in this study are lower than those diffusion coefficients previously observed in single-ion systems. This behavior can be attributed to the presence of Na^+^ ions, which reduces the effective free volume within membrane pores and channels available for divalent ion transport. In similar Na^+^–Ba^2+^ systems, it has been observed that the Na^+^ self-diffusion coefficient remains nearly constant and independent of the mixture composition, whereas the divalent ion diffusion coefficient decreases significantly (by up to 70%) with increasing sodium content in the membrane.^74^

While the results obtained for *D*_Na^+^_ in this study are consistent with the magnitude of previously reported diffusion coefficients, Na^+^ mobility appears to be primarily correlated with membrane hydration and less affected by the presence of divalent ions. This behavior is attributed to the morphology of the hydrophilic channels and pores.^70,71^ Previous studies suggest that Na^+^ preferentially diffuses through water-rich regions of these channels, whereas divalent ions such as Mg^2+^ interact more strongly with fixed sulfonate groups.^71,74,75^ Consequently, unlike the interaction-driven resistance observed for Mg^2+^, Na^+^ mobility is mainly governed by membrane tortuosity.^17,70^

Self-diffusion coefficients of water compositions were calculated and are presented in Figure S6 of the Supporting Information. While theoretical frameworks often treat water as the medium through which ions diffuse rather than as a diffusing entity itself,^76^ the inclusion of water self-diffusion coefficients in this study aims to provide quantitative insights into the structural constraints imposed by the membrane and their influence on ionic transport. The ability of water to diffuse through the membrane decreases significantly compared to water in solution, reflecting the constraints imposed by the membrane’s structure and interactions. These results suggest that water transport depends on the structure of the polymer matrix and the amount of water present in it. Water self-diffusion coefficients are a key measure used to evaluate the performance of a membrane.^77,78^ Likewise, they indicate how water moves through the microstructure and how the different parts of the system are connected.^11,57^ In this way, water content and dynamics affect macroscopic properties such as ionic conductivity. Although the latter is a collective property that depends on the contribution of all ions, the self-diffusion coefficients, in conjunction with the Nernst–Einstein equation, measure the ionic conductivity across the membrane. Accordingly, as λ increases, ions dissociate more readily from sulfonate groups, improving their mobility and, thus, the conductivity of the ions. It is important to note that the Nernst–Einstein equation assumes that ions move independently of each other and excludes correlations between ion motions, known as ion–ion correlations, which can significantly affect the conductivity.^79^

The relationship between hydration level and conductivity also depends on factors such as the size and charge of the ions. As the hydration level increases, water molecules interact with the membrane’s functional groups, facilitating ion mobility through the polymeric network. However, these dynamics can vary based on the specific ions’ interactions. Divalent ions tend to have a higher retention within the polymeric matrix, negatively impacting conductivity. This is because divalent ions establish stronger interactions and exhibit reduced mobility within the membrane compared to sodium ions, which have higher mobility and, thus, higher ionic conductivity. Also, the structural arrangement of the polyelectrolyte in this membrane model plays a crucial role. Divalent ions can alter the conformation of the polymeric network, affecting the distribution and accessibility of ion exchange sites. This change can increase resistance to ion transport, thereby decreasing membrane conductivity. The presence of divalent ions also impacts the structural arrangement of the polyelectrolyte and water dynamics, which may further contribute to increased resistance.

### 3.6 Porous Structure and Hydrophilic Network Formation

Porosity and tortuosity are key parameters that define the internal structure of polymeric materials. The mobility of ions and water molecules increases as porosity rises and decreases as tortuosity increases.^77,80^ Tortuosity characterizes the networks of interconnected channels and pores within the membrane.^77^ It provides information about the membrane’s structure, porosity and ion diffusion. In this study, tortuosity was calculated as a preliminary estimate to evaluate microstructural consistency. To further refine these values and minimize finite-size effects, future research utilizing larger scales and extended time scales is recommended. Moreover, porosity plays a fundamental role in the transport of both water and ions. To control porosity is crucial for improving the design of ion exchange membranes.^11,81^ Porosity directly influences hydrodynamic transport coefficients, electroosmotic entrainment, and water permeation. It also affects ion transport efficiency through the membrane, critical for applications such as RED.

Using the results of *D*_H_2_O_ in the membrane (*D*_H_2_O_^m^) and the local self-diffusion coefficients for water in solution (*D*_H_2_O_^S^), the tortuosity of the membrane was calculated as described in ref 77. The local self-diffusion coefficients were estimated from simulations of a solution with a concentration equivalent to that of the membrane pore solution (see Table S3 in Supporting Information). Figure 9 displays the variation of tortuosity with respect to the water fraction in the system. These results were compared with experimental data on local tortuosity reported for other cation exchange membranes synthesized from poly­(ether sulfone) random copolymers with different degrees of functionalization (20% and 60%).^77^

**Figure 9:**
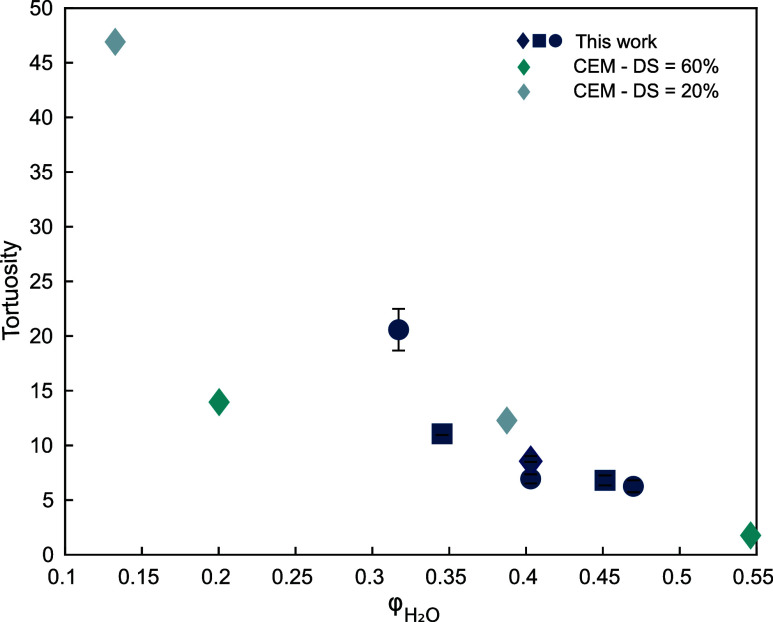
Tortuosity of the polymer matrix for different hydration levels and compositions. In the graph, the different geometrical figures represent the Mg^2+^/Na^+^ concentration ratio for each system: ⧫ (0.0), ■ (0.15) and ● (0.5).

Figure 10 displays the pore limiting diameter (PLD) and the average pore diameter (APD). PLD and APD provide key information for characterizing the morphological structure of the membrane, given that the topological arrangement of the pores influences the transport properties of a porous material. These two parameters describe different structural aspects. On the one hand, the PLD is defined as the diameter of a probe that can percolate through the pore network, corresponding to the narrowest constriction along a continuous diffusion pathway.^82^ In this work, the PLD was calculated using the PoreBlazer package^82^ with a helium probe to identify the smallest accessible bottleneck. On the other hand, the APD is derived from the pore size distribution (PSD) and represents a statistical average over all accessible pore regions.

**Figure 10:**
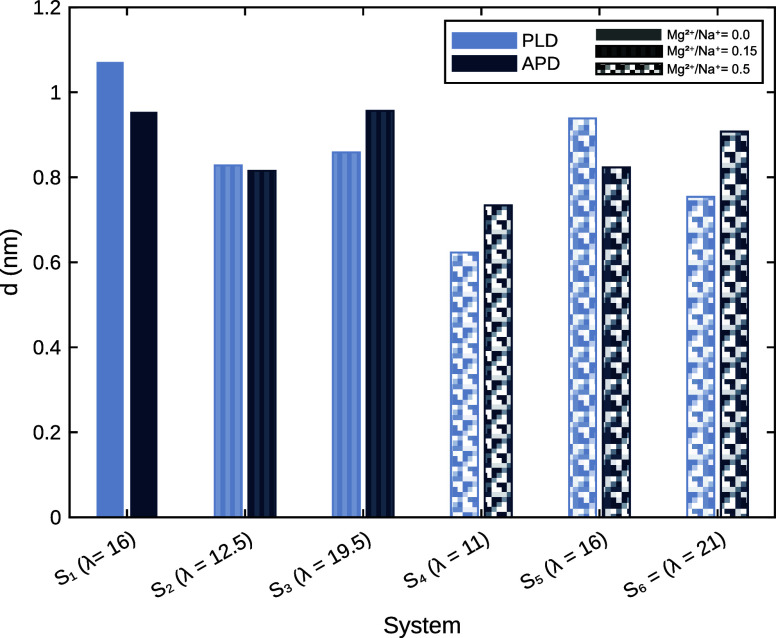
Pore limiting diameter (PLD) and average pore diameter (APD) for different hydration levels and compositions.

The close agreement between PLD and APD values indicates a strong correlation between the capacity of the water domains and their functional connectivity, as shown in prior RDF results. Increasing the hydration level (λ) results in a proportional increase in both the transport channels (PLD) and the total pore volume (APD). This behavior reveals that water content drives the formation of wider and more interconnected water networks, which reduce tortuosity and enhance ion mobility. For instance, at a hydration level of 11, the PLD is 0.623 nm (the smallest among all systems), indicating the most severe structural restriction to diffusion. The comparatively higher APD of 0.734 nm suggests that S_4_ has a notable pore volume that is poorly interconnected due to extremely narrow and highly tortuous regions. By contrast, systems at higher hydration levels (λ = 16–21) display larger PLD and APD values than S_4_, with PLD ranging from 0.754 to 1.071 nm and APD from 0.907 to 0.954 nm, indicating more open and better-connected pore structures. These results are consistent with X-ray scattering spectroscopy experiments, which have observed that the sizes of channels and pores in membranes are typically a few nanometers in diameter.^58^

An increase in Mg^2+^ concentration appears to reduce the pore size of the membrane. Systems with higher divalent ion content (S5 and S6) show lower PLD values (0.939 and 0.754 nm, respectively) and lower APD values (0.823 and 0.907 nm, respectively) compared to system S1 (PLD = 1.071 nm and APD = 0.954 nm), which contains only monovalent ions. This behavior is consistent with the swelling reduction and structural tightening effects reported for polyelectrolyte hydrogels in the presence of divalent cations.^62,83^ The higher charge density of Mg^2+^ promotes electrostatic cross-linking or bridging between polymer chains, limiting the diameter of the functional diffusion pathway.^62,83^

Based on PLD and ADP results, the size of the membrane pores, and thus the interconnected network of channels, is influenced by both water content and ion composition. This result is consistent with the radial distribution functions discussed earlier, which show a smaller spacing of functional groups due to swelling reduction and membrane chains compaction. The swelling reduction effect implies reduced porosity in the polymeric matrix, affecting the minimum and maximum size of the empty spaces or pores within the polymeric network. As the membrane pore size decreases, the tortuosity of the interconnected network of water channels increases. Consequently, divalent ions significantly influence membrane permeability and perm-selectivity. Previous experimental studies have shown that reduced swelling and water uptake in polymeric membranes decrease effective pore size and alter microstructure, leading to changes in intrinsic permeability and selectivity.^84–86^ Additionally, in nanofiltration membranes, the presence of divalent cations has been shown to promote denser packing structures and higher rejection compared to monovalent ions, highlighting the role of ion valence in modulating water transport and enhancing solute rejection.^87^

Finally, this study provides molecular-level insight into ion transport mechanisms relevant for the design of ion-exchange membranes for electrodialysis and reverse electrodialysis applications. The results show that ion transport depends not only on hydration level but also on the strength of specific ion–polymer interactions. Strong coordination between Mg^2+^ and sulfonate groups promotes polymer tightening, reduces effective pore size, and increases transport resistance. These findings indicate that membranes operating in divalent-ion-rich environments should minimize excessive ion–polymer binding. For instance, increased swelling capacity or controlled hydrophobicity can promote weakened Mg^2+^–functional group interactions and increase monomer ion selectivity. In addition, membrane performance can be improved by controlling microstructural features such as porosity and pore connectivity. Tuning membrane porosity can help to control ion mobility and conductivity. This can be achieved through fabrication strategies that regulate pore size distribution, including composite membrane architectures, pore-filled membranes and post-treatment approaches.

## 4 Conclusions

This study demonstrates that hydration and ionic composition significantly impact the transport properties of SPEEK polymeric membranes. Increased hydration enhances the mobility of ions and water molecules by promoting the formation of more interconnected water channel networks within the membrane. As water content rises, the transport properties of ions and water molecules approach those observed in solution. Conversely, the heterogeneous structure of the polymer membrane introduces anomalous diffusion, which impedes ion and molecule transport. Additionally, electrostatic interactions between ions and the polymer, particularly with divalent ions like Mg^2+^, increase the activation energy required for transport, thereby significantly restricting ion mobility. A transition to anomalous diffusion behaviors was observed with decreasing water content and increasing concentrations of divalent ions.

Divalent ions, such as magnesium, affect the polymer structure through strong electrostatic interactions with functional groups and water molecules. These interactions reduce the distance between functional groups, decrease pore size, and increase the tortuosity of hydrophilic channels. Consequently, this effect limits the transport of divalent ions and impacts the transport of water molecules and other ions.

Polymer structure and water content are crucial factors influencing ion transport. Specifically, the shape and tortuosity of hydrophilic domains within the polymer matrix are vital parameters affecting adequate ion mobility. Optimizing the diffusion of monovalent ions is essential to enhance membrane performance in reverse electrodialysis technology. Key factors such as ion size, charge, porosity, and hydration energy should be considered. Engineering the polymer structure to control parameters like porosity and the orientation of binding sites can favor the transport of ions like Na^+^ based on their size, charge, and hydration energy. Furthermore, increasing the membrane’s hydration level boosts the mobility of ions and water molecules. Practically, this highlights the importance of controlling hydration levels to optimize ionic conductivity in SPEEK membranes, considering fabrication protocols and the use of other structural polymers that influence maximum water content, pore size, and distribution.

## Supplementary Material



## Notes

Interaction parameters for water and ions used in molecular dynamics simulations (Tables S1 and S2); equilibration data (Figure S1); analysis of the hydrated polymer structure through radial distribution functions (Figure S2); mean squared displacement plots for ions and water molecules (Figures S3 and S4); anomalous diffusion exponents and self-diffusion coefficients (Figures S5 and S6); and calculated local diffusion coefficients within membrane pores (Table S3) (PDF)

## Abbreviations

MD: molecular dynamics
RED: reverse electrodialysis
SPEEK: sulfonated poly­(ether ether ketone)
MSD: mean squared displacement
RDF: radial distribution function
PLD: pore limiting diameter
APD: average pore diameter
