# Supplementary material for: Molecular Dynamics Study of Magnesium and Sodium Ion Transport in a Sulfonated Cation Exchange Membrane
Source: ACS Omega. 2026 Jul 17;11(29):43459–73. doi: 10.1021/acsomega.6c02058 (PMC13425336; doi:10.1021/acsomega.6c02058)
Supplement: Supplementary file 1 [file ao6c02058_si_001.pdf]

Supporting information

Molecular Dynamics Study of Magnesium and Sodium Ion Transport in a Sulfonated Cation Exchange Membrane

María Pérez-Grisales[1], Iván Moncayo-Riascos[1], Sergio Castañeda[1] and Carlos Sánchez-Sáenz[1]*

1 Departamento de Procesos y Energía, Facultad de Minas, Universidad Nacional de Colombia, Medellín, Antioquia, Colombia

*Corresponding author:

E-mail address: cisanche@unal.edu.co

Interaction parameters for water and ions used in molecular dynamics simulations

Table S*1*. Parameters of the SPC/E interactions model and the Lennard-Jones potential for water [1]

Atom	$\varepsilon\left(\frac{kJ}{mol}\right)$	$\sigma(nm)$	$q\ (e)$	$r\ (nm)$	$K_{bonds}\ \left(\frac{kJ}{mol}\right)$	$HOH\ (deg)$	$K_{angles}\ \left(\frac{kJ}{mol}\right)$
O	0.650	0.3166	- 0.8476	0.1	2510.4	109.47	313.8
H	-	-	0.4238				

Table S*2*. Parameters of the Lennard-Jones potential for ions

Atom	$\varepsilon\left(\frac{kJ}{mol}\right)$	$\sigma(nm)$	$q\ (e)$	Ref
Na^{+}	1.475	0.21595	1.0	[2]
Mg^{2+}	3.6	0.163	2.0	[3]
Cl^{-}	0.0535	0.48305	-1.0	[2]

Equilibration

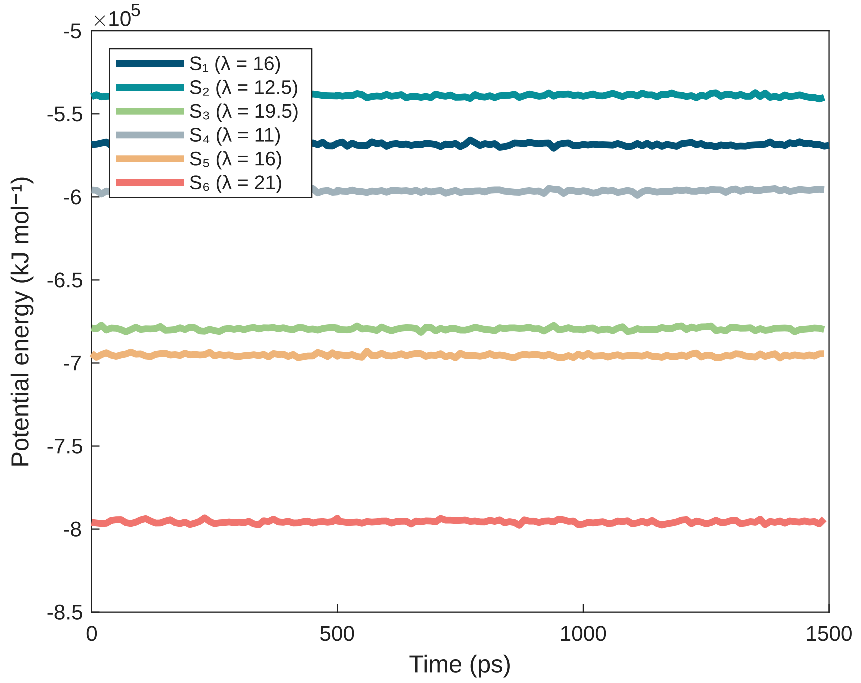

Figure S1. Variation of potential energy during the last 1500 ps of the equilibration step, corresponding to the NVT assembly.

Structure of the hydrated polymer

To examine the final structure of the hydrated polymer, the radial distribution functions between the relevant atoms were contrasted with previously published data in the literature. The RDFs were obtained for the sulfur atoms of the functional groups (S−S), the sulfur atom of the functional group interacting with the water molecules ($S-O_w$), and finally, for the oxygen atom with the partial negative charge of the functional group and the water molecules (O_s-O_w) (see Figure S2).

On the one hand, the dominant peak in the RDFs between $S-O_w$ is associated with the first solvation layer of the main functional groups (see Figure A-S1). In this work, it reaches a maximum value of 3.49 at a distance of 0.395 nm. Bahlakeh et al. [4] reported a first peak at a similar position, approximately 0.3625 nm, with an average height of 2.5. Also, the authors considered a slightly higher water content (λ = 13–14.8) than this work (λ = 11) [4]. The differences in water content and system size may explain the variations observed in the RDFs of both systems. The intensity of the $S - O_w$ peak decreases as hydration increases, which is attributed to the enhanced solvation effect at higher water content, reducing interactions between water molecules and sulfur atoms. Despite these differences in polymer chain length, hydration level, and sulfonation degree, the overall hydration shell structure remains similar. On the other hand, in Figure B-S1 the RDF for O_s-O_w exhibits a peak at 0.265 nm, consistent with previously reported results for comparable systems reported by Bahlakeh et

al. [5] and Chen et al. [6]. At this distance, the oxygen atom of the sulfonate functional group is, on average, coordinated with two water molecules.

Finally, the RDF for S−S was compared with the results reported by Bahlakeh et al. [4] (see Figure C-S1). In this work, the dominant peak in the S−S RDF appears at 0.66 nm, whereas in the work reported by the authors, it is located at 0.539 nm. This displacement is consistent with the expected structural reorganization that occurs at higher degrees of sulfonation. An increase in water content enhances solvation effects and strengthens electrostatic repulsion between sulfonate groups [4], [5]. Furthermore, the height of the $g(r)_{S-S}$ RDF peak in this study is 1.4, closely matching the value of 1.3 reported by Bahlakeh et al. in their study [5]. This similarity suggests that, despite differences between both systems, such as the degree of sulfonation, water content, system size, and the presence of SPES in their system, the spatial correlation between sulfonate groups remains comparable.

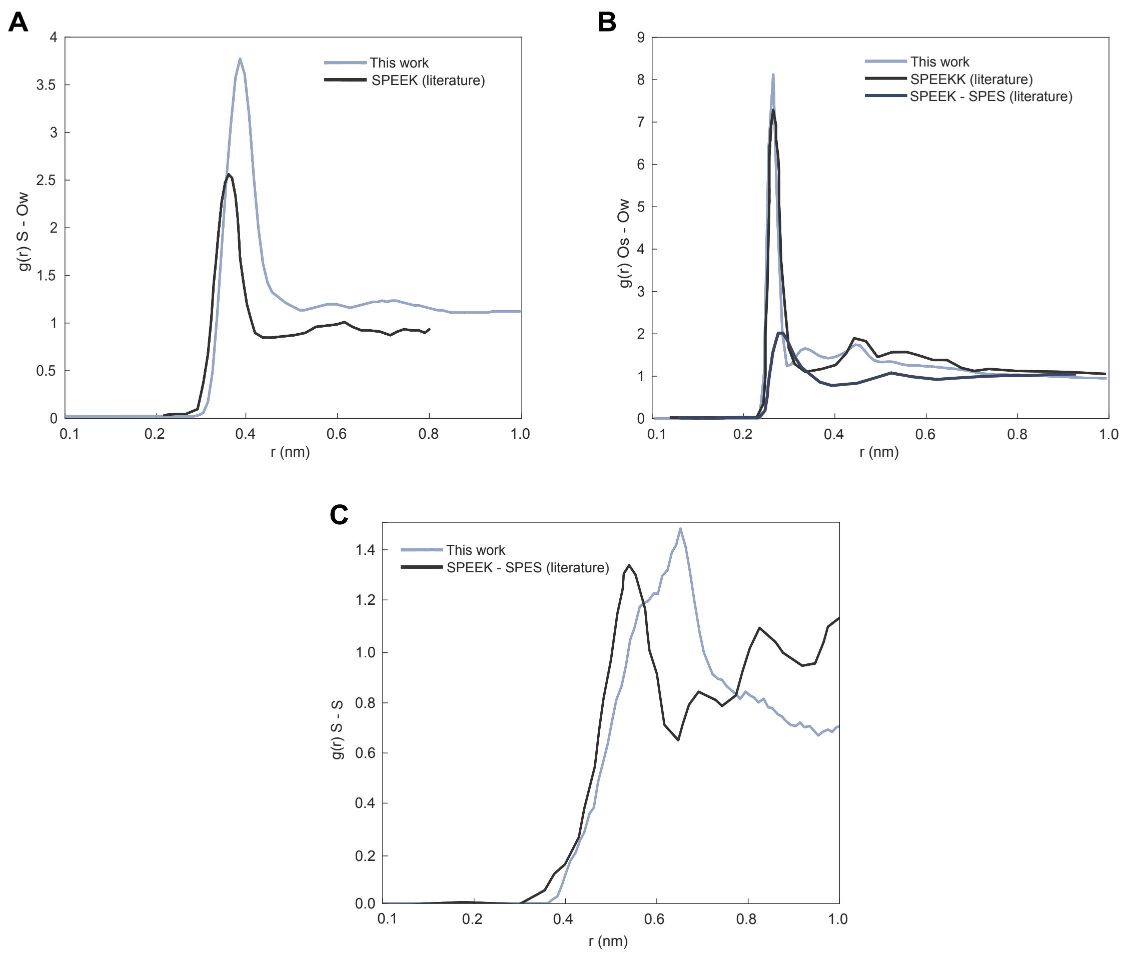

Figure S2. RDFs for λ = 11. A) Sulfur (S) atoms from the sulfonic acid groups and oxygen (O_W) atoms from the water molecules. The obtained results are compared with literature data for SPEEK (62% SD, λ = 13) [4]. Comparison of the oxygen (O_S) atoms from the sulfonic acid groups and O_W with previous results reported for SPEEKK [6] and for a polymer membrane based on a 50:50 (w/w) blend of SPEEK (60% SD) and SPES (40% SD) at λ = 12 [5]. C) Sulfur–sulfur (S–S) atoms from the sulfonic acid groups, compared with results reported for the SPEEK–SPES system [5].

Mean squared displacement of ions and water molecules

Figure S2 shows the MSD plots for sodium and magnesium ions in each simulation and Figure S3 displays the MSD plots for water.

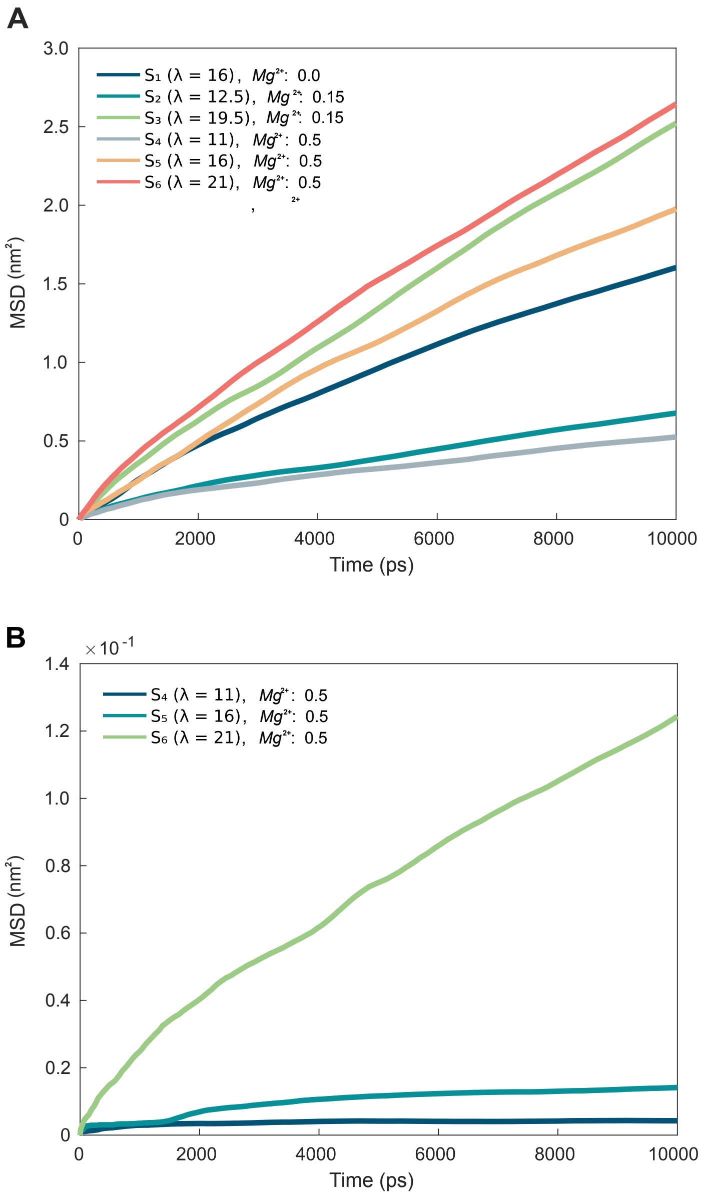

Figure S3. MSD plots for each system where the hydration level and composition vary. A) MSD(t) for the sodium ion. B) MSD(t) for magnesium ion.

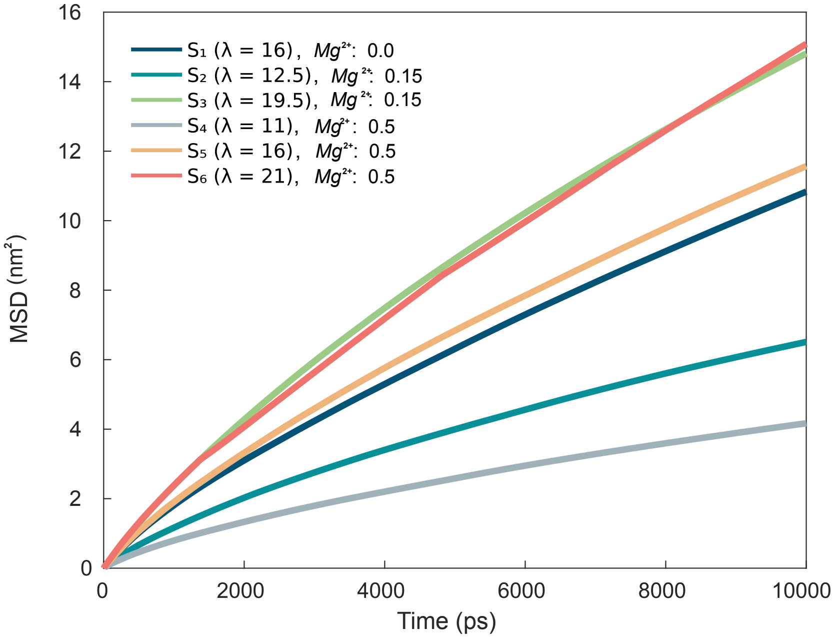

Figure S4. MSD plots of water molecules for each system with different hydration levels and composition.

Anomalous diffusion exponent for ions and water molecules in the membrane

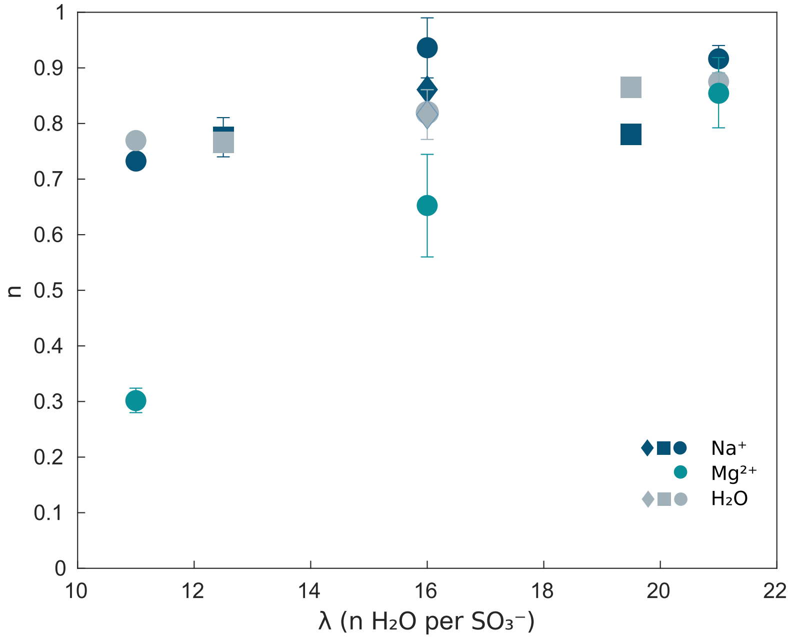

Figure S5. Anomalous diffusion exponent (n) of water molecules and cations. In the graph, the different geometrical figures represent the Mg^{2+}/Na^+ concentration ratio for each system: ♦ (0.0), ■ (0.15) and ● (0.5).

Self-diffusion coefficient of water molecules

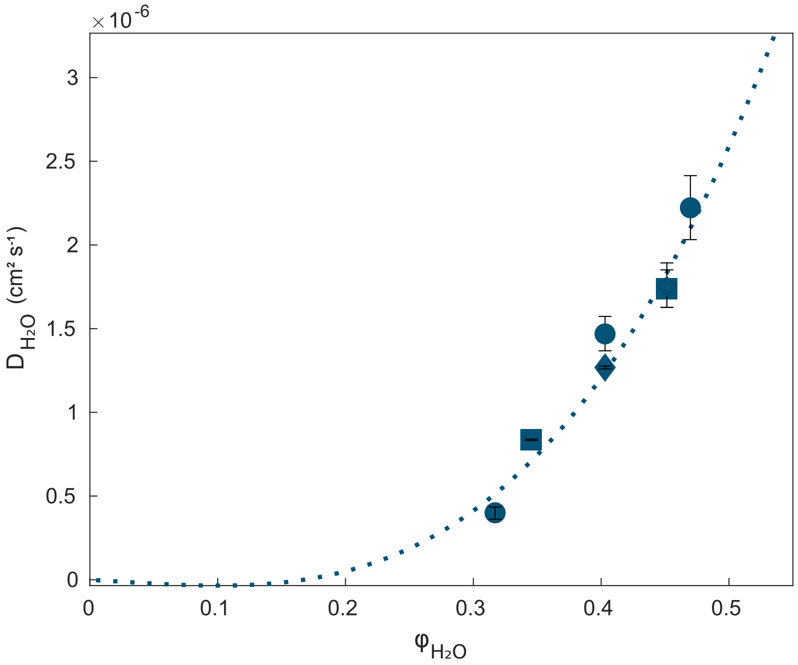

Figure S6. Effective self-diffusion coefficient of water molecules. In the graph, the different geometrical figures represent the Mg^{2+}/Na^{+} concentration ratio for each system: ♦ (0.0), ■ (0.15) and ● (0.5).

Simulations of the solution within the pores

To calculate the local water self-diffusion coefficients (D_{H2O}^{S}), simulations were performed for solutions with ion concentrations equivalent to those found within the membrane pores for each system. These simulations aimed to mimic the local environment of the pores in the membrane, ensuring that the composition of ions was consistent with the conditions in each system. The resulting local self-diffusion coefficients of water are presented in Table S3.

Table S*3*. Water self-diffusion coefficients in solution within the membrane pores.

System	$\boldsymbol{D \times 10^{-9} m^2 s^{-1}}$	$\boldsymbol{Standard\ error}$
S1	1.084	0.001
S2	0.928	0.014
S3	1.187	0.001
S4	0.819	0.005
S5	1.031	0.002
S6	1.393	0.019

References

[1] M. Agarwal, M. P. Alam, and C. Chakravarty, "Thermodynamic, diffusional, and structural anomalies in rigid-body water models," *The Journal of Physical Chemistry B*, vol. 115, no. 21, pp. 6935–6945, 2011.

[2] G. Lanaro and G. Patey, "Molecular dynamics simulation of NaCl dissolution," *The Journal of Physical Chemistry B*, vol. 119, no. 11, pp. 4275–4283, 2015.

[3] S. Mamatkulov, M. Fyta, and R. R. Netz, "Force fields for divalent cations based on single-ion and ion-pair properties," *The Journal of Chemical Physics*, vol. 138, no. 2, 2013.

[4] G. Bahlakeh, M. Nikazar, M.-J. Hafezi, E. Dashtimoghadam, and M. M. Hasani-Sadrabadi, "Molecular dynamics simulation study of proton diffusion in polymer electrolyte membranes based on sulfonated poly(ether ether ketone)," *International Journal of Hydrogen Energy*, vol. 37, no. 13, pp. 10256–10264, 2012.

[5] G. Bahlakeh, M. Nikazar, and M. M. Hasani-Sadrabadi, "Understanding structure and transport characteristics in hydrated sulfonated poly(ether ether ketone)–sulfonated poly(ether sulfone) blend membranes using molecular dynamics simulations," *Journal of Membrane Science*, vol. 429, pp. 384–395, 2013.

[6] P. Chen, C. Chiu, and C. Hong, "Molecular structure and transport dynamics in Nafion and sulfonated poly(ether ether ketone ketone) membranes," *Journal of Power Sources*, vol. 194, no. 2, pp. 746–752, 2009.
